# Lead Contamination in Selected Foods from Riyadh City Market and Estimation of the Daily Intake

**DOI:** 10.3390/molecules15107482

**Published:** 2010-10-25

**Authors:** Zeid A. Al Othman

**Affiliations:** Department of Chemistry, College of Science, King Saud University, Riyadh 11451, Saudi Arabia; E-Mail: zaothman@ksu.edu.sa; Tel.: +96614675999, Fax: +96614675992

**Keywords:** lead contamination, food basket samples, lead daily intake

## Abstract

This study was carried out to determine lead contamination in 104 of the representative food items in the Saudi diet and to estimate the dietary lead intake of Saudi Arabians. Three samples of each selected food items were purchased from the local markets of Riyadh city, the capital of Saudi Arabia. Each pooled sample was analyzed in triplicate by ICP-AES after thorough homogenization. Sweets (0.011–0.199 μg/g), vegetables (0.002–0.195 μg/g), legumes (0.014–0.094 μg/g), eggs (0.079 μg/g), meat and meat products (0.013–0.068 μg/g) were the richest sources of lead. Considering the amounts of each food consumed, the major food sources of lead intake for Saudi can be arranged as follows: vegetables (25.4%), cereal and cereal products (24.2%), beverages (9.7%) sweets (8.2%), legumes (7.4%), fruits (5.4%) milk and milk products (5.1%). The daily intake of lead was calculated taking into account the concentration of this element in the edible part of the daily consumption data which were derived from two sources, (a) the KSA food sheet provided by the Food and Agriculture Organization (FAO) and (b) from questionnaires distributed among 300 families in Riyadh city. The results showed that the daily intakes of lead according to the two sources are 22.7 and 24.5 μg/person/day respectively, which are lower than that mentioned by The Joint Expert Committee on Food Additives (JECFA), whereas it is comprabale with that of other countries.

## 1. Introduction

Lead is a toxic metal that can be found everywhere in the environment. Overexposure to lead continues to be an important worldwide problem [1]. Food is an important source of lead and determination of lead in food can be used for the estimation of lead exposure [2]. The level of lead in the Earth’s crust is about 20 μg/g. The Industrial Revolution gave rise to an increase in the amount of lead in the environment and an even bigger increase occurred around 1920 when leaded gasoline was introduced. Leaded gasoline is still not banned everywhere in the World, and it still used in the developed countries. In areas where leaded gasoline is banned, the major exposure pathways of non- smoking adults are from food and water. Several studies were done to determine the concentration of lead in foods [3,4,5] and to study its dangerous effects. Recently, the USA, all European countries and many developing countries have outlawed or strictly regulated the use of leaded petrol. In such countries, levels of lead in food and drinking water are closely monitored. Lead may reach and contaminate plants, vegetables, fruits and canned food through air, water and soil during cultivation [5,6,7] and also during industrial processing and packaging [8]. Fruits and vegetables grown in polluted soils may become contaminated as a result of plant uptake of lead from soils or direct deposition of leaded dust onto plant surfaces [9,10]. Therefore, through these diverse mechanisms, lead deposited in soil becomes a persistent and long-term source of lead exposure for humans.

Lead exposure can occur through food, water, soil, air and the relative contributions from individual sources may depend on lifestyle and socioeconomic status. It was reported that the main sources of exposure for an adult are food (ranging from 0.4 μg/kg bw/week to 10.1 μg/kg bw/week) and water (ranging from 0.23 μg/kg bw/week to 0.35 μg/kg bw/week). The main potential sources of exposure to lead in children are food, air, water and dust or soil. It has been reported that the dietary intakes of children (ranging from 0.6–30 μg/kg bw/week) could be two to three times that of adults. In other dietary exposures studies such as those conducted in the UK and mainland China, cereals and vegetables were found to be the main dietary sources of lead, which contributed 31% to 40% of total dietary exposure for cereals, and 23% to 35% of total dietary exposure for vegetables [11,12].

Watanabe *et al*. [13] reported that people, specially those who consume rice as a staple food for daily energy, are inevitably exposed to significant amount of lead via rice. Rice crops, even from non- polluted areas, may be contaminated because of fertilizers that are used in forms containing lead. Amongst other foods fish are constantly exposed to lead from polluted water. Lead accumulates in their bodies tissues in different amounts depending on the size and age of fish [14,15,16]. Canned fish, especially canned tuna fish, is abundantly consumed because of its convenient and affordable use. In the same respect, several studies [17,18,19] have reported that the lead content in different type of chocolates varied between 0.07 and 4.0 μg/g product. 

The United Nations World Health Organization (WHO) has also given recommendations for maximum lead content in food and water [20,21]. The Joint FAO/WHO Expert Committee on Food Additives (JECFA) [22] has established a provisional tolerable weekly intake (PTWI) of 25 μg/kg bw/ week for lead. PTWI is an estimate of the amount of a contaminant that can be ingested over a lifetime without appreciable risk. An intake above the PTWI does not automatically mean that health is at risk. Transient excursion above the PTWI would have no health consequences provided that the average intake over long period is not exceeded as the emphasis of PTWI is a lifetime exposure. Developing countries have been slow in putting in place regulatory restrictions on leaded products, and leaded petrol is still used in some cases. There is very little data on the magnitude of the contamination levels of lead in environment and in food in these countries.

Lead can be very harmful even at low concentrations when ingested over a long time [23,24,25]. After ingestion, the absorption rate of lead ranges from 3% to 80%, whereas the typical absorption rates of dietary lead in adults and infants are 10% and 50%, respectively. After absorption, lead is initially distributed to soft tissues throughout the body via blood, and then deposited in bone. Lead is excreted through the kidney and to a lesser extent in the bile while non-absorbed dietary lead is excreted in the feces. Organic lead may be metabolized to inorganic lead. The concentration of lead in blood is commonly used as a biomarker of exposure. Lead is a classical chronic toxic chemical. It may cause damages to kidneys, the cardiovascular, immune, hematopoietic, central nervous and reproductive systems. Short term exposure to high level of lead can cause gastrointestinal distress, anemia, encephalopathy and death [26]. The effect of exposure to lead varies according to dose and the age of the exposed person [27]. Manifestations of lead poisoning are nonspecific abdominal pain, constipation, irritability, myalgia, muscle aches, headache, anorexia, and decreased lipid concentrations. The Kingdom of Saudi Arabia (KSA) imports a lot of foodstuffs from several countries. These foods may be subjected to lead contamination as described above. The objective of this work was to estimate the levels of lead that may be present in some foods available in local markets in Riyadh city, to determine the food daily intake from the Food Consumption Survey (questionnaire) conducted in local population in Riyadh city 2010, and consequently the daily lead intake from food (questionnaire) and finally, to compare lead intake from the questionnaire with that from the food balance sheet of the KSA provided by the FAO.

## 2. Results

The data in Table 1 shows the measured lead content in various food items. The number of selected food items in each food group was as follows: cereals and cereal products (n = 9), legumes (n = 8), vegetables (n = 34), pickles (n = 2), fruits (n = 9), meats and meat products (n = 11), sweets (n = 10), eggs (n = 1), fat and oils (n = 4), beverages (n = 9). The highest lead content were found in sweets (0.199 μg/g Snickers, 0.131 μg/g Triplex, 0.092 μg/g Twix, 0.083 μg/g Galaxy), 0.094 μg/g cowpeas, the medium concentration were found in egg (0.074 μg/g), chicken (0.068 μg/g), sardines (0.063 μg/g) while the lowest concentrations were found in bears and time (0.002 μg/g of each) and potato (0.003 μg/g).

**Table 1:** Concentration of lead in analyzed food samples.

Food kind	No	Sample	Mean lead concentration (μg/g dry weight food)
**Vegetables**	1	Cucumber	0.016 ± 0.009
	2	Zucchini	0.013 ± 0.011
	3	Green peas	0.005 ± 0.003
	4	Carrot	0.027 ± 0.015
	5	Potatoes	0.003 ± 0.001
	6	Eggplant	0.024 ± 0.019
	7	Green peppers	0.012 ± 0.0011
	8	Okra	0.025 ± 0.021
	9	Time	0.002 ± 0.001
	10	Green hot pepper	0.024 ± 0.017
	11	Sweet Potato	0.013 ± 0.06
	12	Taro	0.025 ± 0.009
	13	Onion	0.015 ± 0.011
	14	Garlic	0.012 ± 0.002
	15	Canned taro	0.023 ± 0.013
	16	Canned green peas	0.011 ± 0.007
	17	Canned carrots	0.025 ± 0.012
	18	Canned okra	0.038 ± 0.016
	19	Canned Spinach	0.007 ± 0.003
	20	Canned Potato	0.041 ± 0.023
	21	Canned jews mallow	0.020 ± 0.0016
	22	Mallow	0.014 ± 0.009
	23	Purslane	0.004 ± 0.002
	24	Radish	0.014 ± 0.011
	25	Dill	0.027 ± 0.015
	26	Spinach	0.015 ± 0.008
	27	Lettuce	0.033 ± 0.023
	28	Round leafy lettuce	0.195 ± 0.065
	29	Mint	0.020 ± 0.012
	30	Coriander	0.015 ± 0.009
	31	Cauliflower	0.005 ± 0.002
	32	Watercress	0.061 ± 0.027
	33	Cabbage	0.027 ± 0.008
	34	Green onions	0.027 ± 0.009
**Fruits**	35	Tomatoes	0.025 ± 0.017
	36	Banana	0.013 ± 0.007
	37	Strawberry	0.017 ± 0.009
	38	Orange	0.013 ± 0.004
	39	Pears	0.002 ± 0.001
	40	Lemon	0.014 ± 0.003
	41	Apple	0.019 ± 0.005
	42	Canned strawberry	0.030 ± 0.011
	43	Dried dates	0.018 ± 0.009
**Pickles**	44	Pickled cucumber	0.023 ± 0.02
	45	Pickled turnip	0.027 ± 0.018
**Legume**	46	Green kidney Bean	0.055 ± 0.031
	47	Canned green kidney beans	0.047 ± 0.025
	48	Canned kidney beans	0.018 ± 0.009
	49	Canned beans	0.019 ± 0.006
	50	Canned beans	0.031 ± 0.015
	51	Beans	0.014 ± 0.002
	52	Canned peas	0.048 ± 0.029
	53	Cowpeas	0.094 ± 0.033
**Cereal and cereal products**	54	Pasta	0.047 ± 0.013
	55	Rice (Karos Snowight)	0.032 ± 0.021
	56	Rice (Abu Sion)	0.020 ± 0.01
	57	Bread	0.015 ± 0.009
	58	White bread	0.008 ± 0.001
	59	Biscuits (Tyoshob)	0.025 ± 0.015
	60	Biscuits (Tyoshob strawberry)	0.018 ± 0.01
		Tea Biscuits	0.036 ± 0.022
	62	Biscuits (Loucker)(Napolitaner)	0.032 ± 0.015
**Milk and Milk Product**	63	Fresh milk	0.006 ± 0.003
	64	Danette banana-flavored milk	0.018 ± 0.008
	65	Danette strawberry-flavored milk	0.027 ± 0.012
	66	Yogurt	0.025 ± 0.01
	67	Cheese	0.003 ± 0.001
	68	Ice Cream (plastic tray)	0.055 ± 0.024
	69	Ice Cream (paper package	0.014 ± 0.01
	70	Vanilla ice cream (paper package)	0.032 ± 0.021
**Beverages**	71	Orange and carrot juice	0.016 ± 0.011
	72	Apple juice	0.014 ± 0.009
	73	Fruit juice	0.050 ± 0.041
	74	Orange juice	0.010 ± 0.009
	75	Lemon juice	0.053 ± 0.026
	76	Strawberry juice	0.016 ± 0.011
	77	Lipton tea packages	0.046 ± 0.029
	78	Lipton Tea	0.065 ± 0.033
	79	Coffee	0.053 ± 0.041
**Sweets**	80	Bounty	0.051 ± 0.034
	81	Galaxy	0.083 ± 0.045
	82	Snickers	0.199 ± 0.04
	83	Vip	0.066 ± 0.019
	84	Albeni	0.021 ± 0.008
	85	Triplex	0.131 ± 0.004
	86	Towers gold	0.044 ± 0.024
	87	Twix	0.092 ± 0.032
	88	Rush	0.011 ± 0.008
	89	Halvah	0.080 ± 0.02
**Meat and meat products**	90	Sardines Haakon hot	0.050 ± 0.01
	91	Light meat tuna (Eldiafa)	0.049 ± 0.027
	92	Light meat tuna (C Harvest)	0.025 ± 0.013
	93	Cooked fish (Incheon Rural Slides)	0.013 ± 0.009
	94	Light meat tuna (IFFCO)	0.042 ± 0.018
	95	Light meat tuna (California)	0.039 ± 0.022
	96	Sardines cooked (Milo)	0.063 ± 0.015
	97	Chicken	0.026 ± 0.018
	98	Fish	0.068 ± 0.051
	99	Fresh meat	0.042 ± 0.004
	100	Eggs	0.074 ± 0.009
**Fats and oils**	101	Noor	0.020 ± 0.012
	102	Shams	0.030 ± 0.005
	103	Abu Zahra	0.006 ± 0.001
	104	Afia	0.005 ± 0.001

Considering the amount of food consumed, the major food sources of lead intake for Saudis were vegetables (25.4%), cereals and cereal products (24.2%), beverages (9.7%), sweets (8.2%), legumes (7.4%), fruits (5.4%), milk and milk products (5.1%) and finally pickles (1.1%), and the mean daily intake of lead is 24.57 μg/person/day. The lead daily intake for each person was estimated by multiplying the mean intake of each food items by its mean lead content as shown in Table 2.

**Table 2:** Lead daily intake in correlation with the questionnaire.

Kind of food	Daily consumption (g/day)	Pb Conc μg/g	Daily Pb intake μg/person/day	Contribution %
Vegetables	254.49	0.025	6.247	25.4
Fruits	78.02	0.017	1.318	5.4
Pickles	10.39	0.03	0.261	1.1
Legumes	44.91	0.04	1.828	7.4
Cereal and cereal product	270.01	0.022	5.958	24.2
Milk and milk products	83.79	0.01	1.249	5.1
Beverages	66.71	0.04	2.387	9.7
Sweets	54.77	0.04	2.012	8.2
Meat and meat products	108.60	0.029	3.161	12.9
Fats and oils	10.00	0.02	0.153	0.6
Daily lead intake (μg/person/day)			**24.574**	100
Daily lead intake (μg/Kg body wt/day)			0.351	
Lead intake (μg/Kg body wt/week)			2.457	

The results in Table 3 shows the comparison between the estimated daily lead intake for each person from the questionnaire with the calculated daily lead intake from the KSA food balance sheet according to the FAO [28]. As shown from the data in Table 2, the mean daily intake of lead is 24.57 μg/persom/day. This value is similar to the estimated daily intake of lead according to the food balance sheet of KSA of the FAO [28] which was 22.74 μg/person/day (Table 3).

**Table 3:** Lead daily intake in correlation with the KSA food balance given by the FAO.

Food kind	Pb Conc μg/g food	Daily consumption of food (g)	Daily Pb intake μg/person/day
Vegetables	0.025	289.5	7.15
Zucchini	0.013	16.4	0.21
Carrots	0.027	0.26	0.01
Potatoes	0.013	47.6	0.63
Tomatoes	0.02	82.5	2.04
Dry dates	0.02	74.7	1.33
Cereal and cereal product	0.022	332	7.33
White cheese	0.003	50	0.13
Meat and meat products	0.029	135	3.93
Daily lead intake (μg/person/day)			**22.74**
Daily lead intake (μg/Kg body Wt/day)			0.32
Lead intake (μg/Kg body Wt/weak)			2.27

The data in Table 4 show the estimated intake of Pb/person/day. Lead daily intake (24.5 μg/person/day) of Saudi subjects shows a higher value than that of the cities of Seoul and Tainan (21.5 and 19.5 μg/person/day respectively) but considerably lower than that of the cities of Naning and Xian (32.3 and 26.1 μg/person/day respectively). The lowest lead intake per day was recorded in Kualalumpour and Tokyo which were 7 and 9.3 μg/person/day, respectively (Table 4).

**Table 4:** Comparison of the estimated Pb intake of population in various cities.

City	Lead concentration in food (μg/day)	References
**Riyadh**	24.5	Our study
Bangkok	15.1	[64]
Kualalumpur	7	[64]
Manila	11.3	[64]
Tainan	19.5	[64]
Beijing	23.9	[64]
Jinan	26	[64]
Naning	32.3	[64]
Shanghai	17.2	[64]
Xian	26.1	[64]
Tokyo	9.3	[64]
Seoul	21.5	[64]

Ikeda *et al* 2000 [64]

## 3. Discussion

The factors affecting the concentration of lead in food, depends on the type of food, the methods of processing and storage. The food analysis indicated that sweets contain higher amounts of lead than other foods, followed by meat and meat product then by leafy vegetables, while the lowest lead contents were found in fruits and beverages. Processing or storing food in lead contaminated pots causes a significant lead elevation in food. Cooking with acidic foods (such as tomatoes, vinegar, and alcohol or soy source) in these pots is of special concern because their low pH enhances the leaching process [29] for this reason, we found that processed meat and canned legumes contains high amount of lead, as shown in Table 1.

The data in Table 1 shows that some sweets contain higher amounts of lead such as Snickers (0.199 μg/g), Triplex (0.131 μg/g), Twix (0.092 μg/g), Galaxy (0.085 μg/g) and Halvah (0.08 μg/g). Recent warnings issued by the U.S. Food and Drug Administration (FDA) and state health departments have increased concern about contamination in candy imported from Mexico [30,31]. The Centers for Disease Control (CDC) [32] reported that, based on routine blood lead screening, six California children suffered from lead toxicity after eating tamarind- and chili-flavored candy imported from Mexico. The FDA then found between 0.3 μg to 0.4 μg of lead per gram of candy. The California Department of Health Services independently found elevated lead levels in candy as well (>0.5 μg of lead per gram) [31]. It is believed that lead contamination may be introduced from ingredients during candy processing such as drying, storing, and grinding or that lead may leach in to the candy from tainted wrappers [33].

In the same respect, contamination of chocolate by lead is thought to occur because the majority of beans are grown in locations that still use leaded gasoline. Despite high per capita consumption of chocolate in the United States, there is paucity of data of lead concentration in chocolate products. The American Environment Safety Institute, an environmental advocacy group based in California, tested a variety of chocolate products and found that they contained 0.00157 μg and 0.105 μg lead per gram chocolate. The United State Department of Agriculture’s (USDA) total diet study [34] found between 0.0 and 0.11 μg lead per gram chocolate. Analysis of chocolate samples by our research group yielded similar results. Mean lead levels ranged from 0.04 to 0.093 μg lead per gram chocolate except for Triplex and Snickers which had higher levels of lead (0.131 and 0199 μg/g) respectively (Table 1).

The *Codex Alimentarius*, global food standards developed by FAO and WHO, limits the lead content of coca powder or beans to 1 μg of lead per gram product [35]. Analysis of a variety of chocolate products from various global location completed by a Swiss group in 2002, found that the lead content of these items ranged from 0.0011 μg to 0.769 μg/g, below the international standards [36]; this, coupled with research suggesting that only 5% to 10% of lead contained in cocoa is bio- available to the body [37], indicates that there is limited risk of lead toxicity due to to regular consumption of chocolate products.

The maximum lead limit for human health has been established for edible parts of crops as 0.3 μg/g by WHO but this limit in china is 0.2 μg/g. Data in table (1) shows that lead concentration in vegetables, fruits, and cereal products are in the range of 0.002 to 0.192 μg/g. The lead levels in these products are below the maximum WHO or china limits and comparable to those observed in other studies [38]. They attributed the lower levels of lead to the successful phasing out of leaded gasoline in European countries when leaded fuel is burned, it emit very fine particles of lead into the air, where they may settle on vegetables as they are vended along the street and next to bays high ways. Some of particles settle on soil where they later contaminate the food when the dust is blown by wind [39]. Other investigations have also reported high levels of lead content in vegetables sampled near major high ways [40]. Perhaps, not surprisingly, there is good correlation between average traffic counts and average soil and plant lead content of sites close to the road side. An inverse relationship between distance from the road and lead content has been observed in various soil and vegetables [40]. In Japan, Maramatsa, *et al*. [41] found lead concentrations of 2.3 and 2.4 μg/g in spinach and 1.2 μg/g in cabbage.

Yang *et al*. [42] reported that cereal in China had lead levels of 0.06 μg/g. In Denmark the national food agency establishes lead levels of 0.03 μg/g in cereals [43,44]. Another study by Urieta *et al*. [45] found mean lead levels of 0.02 μg/g in cereal from Spain. In the United Kingdom, Ysart [46] reported mean lead levels of 0.02 μg/g in cereal products. In Poland, Krelowska-Kula [47] found lead levels of 0.07 μg/g in cereal. In Japan, Muramatsu *et al*., [41], established lead concentrations of 0.05 μg/g in wheat and rice. While in this study the lead levels in cereal and cereal products ranged from 0.008 μg/g in white bread to 0.047 μg/g in pasta. The lead levels in rice ranged from 0.02 μg/g in Abu Sion rice to 0.032 μg/g in Karose Snowhite Rice.

The maximum acceptable limit of lead levels in fish is 0.2 μg/g according to Turkish and EU limits [48,49]. In this study the lead levels in meat and meat products ranged from 0.013 μg/g in Cooked fish (Incheon Rural Slides) to 0.074 μg/g in eggs, whereas the lead level in cooked sardines was 0.063 μg/g. Sireli *et al*. [50] reported that the concentration of lead in fish samples varied from 0.001 to 0.791 μg/g, with a mean of 0.177 μg/g. Current Turkish legislation specifies the maximum lead limit in fish meat as 0.2 μg/g [48], which are in line with the corresponding EU regulations [49]. Eboh *et al*., [51] reported lead in the muscle, gills and liver tissues of fish at a range of 0.001–0.002 μg/g; on the other hand, the mean concentrations of lead (4.27–6.12 μg/g) reported by Canli and Atli [52] in muscle tissues of six different fish species are higher than our results. The concentration of lead in canned tuna samples were found to be 0.0162–0.0726 μg/g, with a mean of 0.0366 μg/g [53], which are in line with our results. 

In this study the concentration of lead in the fruits drinks ranged from 0.01 to 0.053 μg/g and followed the order lemon > fruit > strawberry > orange and carrot > apple > orange. These concentrations are below the guideline values for lead in juice drinks reported by Chukwujidu *et al*., [54], who found that lead concentration in juice drinks ranged from 0.06 to 1.93 μg/g. The major source of lead in canned fruit drinks is the leaching of lead from the canning process. Adraiano [55] reported lead levels of 0.01 μg/g for beverage drinks in Canadas. Paolo and Maurizio [56] reported mean lead levels of 0.38 μg/g for fruits drinks, while Contrer Aslopez *et al*., [57] reported 0.15 μg/g lead in fruit drinks in Spain. The mean levels of lead in the various brands were below the level reported by these authors except for those reported in Canada by Adraiano [56].

As shown in Table 1 the lead levels in milk and milk products ranged from 0.018 to 0.055 μg/g. These levels are in line with the lead levels in raw milk in samples from various factories in Iran (0.01–0.023 μg/g), whereas the milk lead levels in Poland are very high (1.16–3.74 μg/g), as reported by Baranowska *et al*. [58]. In China the lead concentration levels in milk-based products increased over a period of 10 years [59]. However, although levels of lead in milk seem to have risen slightly in other countries over the past 20–30 years, there is no evidence of any real increase in dietary lead during recent decades. Recent comparisons of lead in food in North-America with those obtained over 20 years earlier also don't suggest any increase in lead intake by man from this source [60].

One of the probable reasons for the increase in other countries may be the wide use of leaded gasoline during recent decades [61]. Another important source of lead contamination in food and feed is water, especially in more contaminated areas [62].

The lead daily intake, extracted from questionnaire daily food consumption was 24.5 μg/person/day. This value is slightly higher than the daily lead intake calculated from the food balance of KSA given by the FAO, which is 22.7 μg/person/day, Table 2 and Table 3. The total lead exposure in European diet (European diet provides the maximum potential for weekly intake of lead through food) would be 4.63 μg of lead per kg body weight [63] for an average person of 60 kg body weight and the mean weekly intake is 277.8 μg, which would be somewhat less than the PTWI of 1,500 μg for lead [22]. However, the lead exposure, especially for infant consumption, should be minimized as much as possible. In this study the weekly intake of lead through food estimated as 171.5 μg/person/week, a value that is much lower than that previously mentioned by JECFA [22].

The data in Table 4 shows that the estimated lead daily intake of the Riyadh population from food (24.5 μg/person/day) is higher than that of the populations of Kuala Lumpur (7 μg/person/day), Tokyo (9.3 μg/person/day), Shanghai (17.2 μg/person/day), Beijing (23.9 μg/person/day) and lower than that of Jinan (26 μg/person/day), Xian (26.1 μg/person/day) and Naning (32.3 μg/person/day) [64].

## 4. Experimental

### 4.1. Sampling

Samples from local foodstuffs from Saudi Arabia (KSA) were collected during May 2009. Three kilograms of each fresh sample were collected from different market in Riyadh city in polyethylene containers to avoid contamination. The groups of foodstuffs studied are shown in Table 5.

**Table 5:** Food samples, name and categories from Riyadh city markets.

Food groups	Samples
Vegetables	Cucumber, zucchini, green peas, carrots, potatoes, eggplant, green peppers, okra, thyme, green hot pepper, sweet potato, taro, onion, garlic, canned taro, canned green peas, canned carrots, canned okra, canned spinach, canned potato, canned jews mallow, mallow, purslane, radish, dill, spinach, lettuce, round leafy lettuce, mint, coriander, cauliflower, watercress, cabbage and green onions
Fruits	Tomatoes, banana, strawberry, orange, pears, lemon, apple, canned strawberry and dried dates
Pickles	Pickled cucumber and pickled turnip
Legumes	Green kidney bean, canned green kidney beans, canned kidney beans, canned beans, canned beans, beans, canned peas and cowpeas
Cereal and cereal products	Pasta, rice (Karos Snowhite), rice (Abu Sion), bread, white bread, biscuits (Tyoshob), biscuits (Tyoshob strawberry), tea biscuits, biscuits, (Loucker Napolitaner)
Milk and milk Products	Fresh milk, Danette banana-flavored milk, Danette strawberry-flavored milk, Yogurt, cheese, ice cream (plastic tray), ice cream (paper package), vanilla ice cream (paper package)
Beverages	Orange and carrot juice, apple juice, fruit juice, orange juice, lemon juice, strawberry juice, Lipton tea packages, Lipton Tea, coffee
Sweets	Bounty, Galaxy, Snickers, Vip, Albeni, Triplex, Towers gold, Twix, Rush Halvah
Meat and meat products	Sardines Haakon hot, light meat tuna (Eldiafa), light meat tuna (C Harvest), Cooked fish (Luncheon Rural Slides), light meat tuna (IFFCO), light meat tuna (California)sardines cooked (Milo), chicken, fish, fresh meat and eggs.
Fats and Oils	Shams, Noor, Abu Zahra and Afia

### 4.2. Sample preparation

For processed foods, those brands commanding a high share of the domestic market were purchased. Three samples for each food item were purchased in May 2009. The samples were dried at 100 °C till constant weight and ground with special mills, with provisions to prevent contamination (grinding parts made of aluminum and titanium), into a homogenous matter. A sample (150 g) was taken and stored in air-tight polyethylene bottles at 18 °C until analysis, as described [65,66].

### 4.3. Chemical analysis

High purity chemicals were used in all tests. All reagents met the specifications of the Committee on Analytical Reagents of the American Chemical Society, and included: nitric acid (ultra pure HNO_3_, 70%) from Wenlab (UK); hydrochloric acid (36%), sulfuric acid, hydrofluoric acid and perchloric acid from BDH Laboratory Supplies (UK); hydrogen peroxide from Petrochem (UK) and reagent water (high purity, 18 MΩ). Food samples was wet digested using heating (150 °C) on a hot plate (PC-351, Corning Incorporated Life Sciences, Lowell, MA, USA) using different acids for each food category according to the methods mentioned in [54,67,68,69]. The same pretreatment procedure was repeated until the darkness of solution disappeared. After cooling, the digested solution was diluted with deionized water and transferred to a 50-mL volumetric flask. The diluted solution was filtered through a filter paper into a polyethylene tube (50 mL), then the lead concentration in the digested samples was determined in ppb using ICPAES [Thermo Scientific ICAP 6500 ICP-AES with SIK (standard introduction kit); glass concentric nebulizer /cyclonic spray chamber, USA] [70]. A recovery study of the analytical procedure was carried out by spiking and homogenizing several already analyzed samples with varied amounts of standard solutions of lead. The spiked samples were processed for the analysis by same procedure as described above. Average recovery obtained was 97.55 ± 1.6.

### 4.4. Estimation of dietary lead intake

The lead intake per person per day was estimated from the KSA food balance sheet given by the FAO and from a questionnaire which was distributed among three hundred families in Riyadh city. The answers on the quantities of food they consumed independentally of financial situation was collected and the mean values were calculated by multiplying the amount of each food categories consumed by the mean lead concentration in each categories, as shown in Table 6.

**Table 6:** Example for lead daily intake calculation from food samples.

Item	Consumption g/day	Mean lead concentration (μg/g)	Daily intake
Vegetables	200	0.025	5
Meat and meat products	100	0.029	2.9
Fruits	150	0.017	2.55
Legumes	100	0.04	4
Cereal and cereal products	200	0.022	4.4
Beverage	100	0.04	4
Sweets	70	0.04	2.8
Daily lead intake ug/person/day			25.65

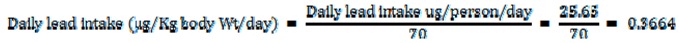
Assuming that body weight is 70 kg

### 4.5. Statistical analysis

The obtained results were analyzed using the SPSS program (version 17).

## 5. Conclusions

By analyzing the lead content in representative food items in Riyadh city, the capital of Saudi Arabia, a lead database was made on a limited scale for 104 food items. The food balance sheet of KSA provided by the FAO was analyzed to select food for analysis. Vegetables, cereals and cereal products, beverages and sweets turned out to be the major source of lead for Saudis. The main daily intake of lead is 24.57 μg/person/day, as calculated from questionnaire data and is 22.7 μg/person/day as calculated from the KSA food balance sheet according to the FAO.
